# Culture-Related Health Disparities in Quality of Life: Assessment of Instrument Dimensions Among Chinese

**DOI:** 10.3389/fpubh.2021.663904

**Published:** 2021-06-09

**Authors:** Minghui Li, Zhang Bao, Gang Lv, Jianying Zhou, Pingyu Chen, Nan Luo

**Affiliations:** ^1^Department of Clinical Pharmacy and Translational Science, University of Tennessee Health Science Center, Memphis, TN, United States; ^2^Department of Respiratory and Critical Care Medicine, The First Affiliated Hospital, College of Medicine, Zhejiang University, Hangzhou, China; ^3^Department of General Surgery, The First Medical Center of Chinese PLA General Hospital, Beijing, China; ^4^Department of Health Economics, China Pharmaceutical University, Nanjing, China; ^5^Saw Swee Hock School of Public Health, National University of Singapore, Singapore, Singapore

**Keywords:** health-related quality of life, culture, health disparities, primary care, Chinese

## Abstract

**Background:** Health-related quality of life (HRQoL) is one of the major focuses of primary care. However, HRQoL instruments used in China are mainly developed from Western countries. Such instruments may not cover all important health concepts valued by the Chinese as health is a culture-specific concept.

**Objectives:** The objectives of this study are to identify culture-specific health dimensions and culture-related health disparities in primary care that are considered important by Chinese living in China.

**Methods:** A purposive sample of 164 adult Chinese (67 healthy persons and 97 patients) were interviewed face to face. In-depth open-ended questions were asked to elicit culture-specific dimensions of quality of life in primary care settings in China.

**Results:** Twelve health dimensions were identified. Five most frequently mentioned dimensions were: mood (*N* = 52, 31.71%), physical activities (*N* = 48, 29.27%), work (*N* = 40, 24.39%), diet (*N* = 32, 19.51%), and vitality (*N* = 28, 17.07%). Significantly more healthy persons reported mood (49.25 vs. 19.59%, *P* < 0.001), mindset (16.42 vs. 0.00%, *P* < 0.001), and self-care (11.94 vs. 2.06%, *P* = 0.016) characterizing good HRQoL, while more patients emphasized on work (4.48 vs. 38.14%, *P* < 0.001). Diet and vitality appeared to be culture-specific dimensions related to health among Chinese.

**Conclusions:** To better adapt or develop HRQoL instruments for Chinese, dimensions or items regarding diet might be included and disparities in the meaning of vitality between Chinese and Western cultures should be considered.

## Introduction

Health-related quality of life (HRQoL) is one of the major focuses of primary care. However, generic and disease-specific HRQoL instruments used in China are mainly developed from North America and Europe. Such instruments, after careful translation and cultural adaptation, usually work well in Chinese in terms of respondents' acceptability and psychometric properties. However, as the concept of health or HRQoL may not be the same across different cultures, these instruments might not be adequate for Chinese people (1–5). The working definitions of health or HRQoL used by Western-originated instruments may not be optimal to Chinese. If this is the case, optimal measurements cannot be achieved with such instruments among Chinese. To the best of our knowledge, no empirical research has been done to assess the adequacy of Western-originated instruments for measuring the Chinese population in primary care settings.

This study aimed to elicit the dimensions that Chinese people think should be used to define health in primary care settings. By knowing such information, we can then assess how valid current instruments are for measuring the HRQoL of the Chinese population and will have a better idea about how to improve HRQoL measurements in the setting of Chinese culture.

## Methods

Data used in the qualitative study were obtained from a survey of healthy persons and patients in China. Both healthy and ill subjects were included for a more comprehensive investigation. Healthy persons were recruited using a quota sampling method and patients were recruited using a convenience sampling method from primary care settings. In the survey, the interviewer asked each subject the following two open-ended questions: “what is your best imaginable health state like?” and “what are the characteristics of good quality of life in your own opinion?” Answers to these questions were summarized as individual themes and recorded by the interviewers.

The themes derived from the two surveys were collated and reviewed independently by two HRQoL researchers. In the review, similar or closely related themes were grouped to form dimensions of health. Only those health dimensions mentioned by more than two participants were reported. The two reviewers then met to reconcile the dimensions they identified and the components for each dimension. After complete consensus was reached, dimensions alluded by each respondent were enumerated and the frequency of each health dimension was compared between the two groups of respondents (i.e., healthy and ill) using Chi-square or Fisher's exact test. Within each group, the association between dimensions and demographics including age ( ≤ 50 vs. >50 years), gender, and education level (high school or lower vs. college or higher) was examined for the most frequently nominated five health dimensions using Chi-square or Fisher's exact test.

## Results

A total of 67 healthy persons and 97 patients were successfully interviewed. Females accounted for 52.2 and 40.2% of healthy and ill participants, respectively. Age groups were evenly distributed among healthy persons; while more patients were older than 50 years (40.2%). The distribution of education level was even among patients; while 50.8% of healthy persons had a bachelor's or higher degree. Most of the participants were full-time employees (47.8% of healthy person and 57.7% of patients).

Twelve health dimensions were identified, including physical activities, leisure activities, work, self-care, diet, sleep, pain, mindset, mood, vitality, cognition, and relationship (Table 1). These health dimensions covered physical, mental, and social health.

**Table 1:** Identified health dimensions from healthy persons and patients.

**Component**	**Dimension**	**Meaning**	**Example component terms**
Physical health	Physical activities	Ability to perform physical activities	Walk, Usual activities, Physical exercise (e.g., run, climb, and play basketball)
	Leisure activities	Ability to perform leisure activities	Play (e.g., dance, travel, and sing Karaoke)
	Work	Ability to work	Work, farm work, housework
	Self-care	Ability to take care of myself	Take care of myself, do not need help from others
	Diet	Ability to eat or drink well	Eat, drink, good appetite, alcohol consumption
	Sleep	Ability to have a sound sleep	Sleep, sound sleep, high-quality sleep
	Pain	Free from pain	No pain, no discomfort
Mental health	Mindset	Positive attitude	Positive attitude, healthy attitude, optimistic attitude, forgiving attitude
	Mood	Good mood	Good mood, happy mood, no worry, no pressure
	Vitality	High vitality	High spirit, no fatigue, have strength
	Cognition	Good cognition	Sharp mind, clear mind
Social health	Relationship	Good relationship with others	Happy family, good relationship with friends, good relationship with others

Healthy persons and patients nominated almost the same dimensions, except for mindset (Table 2). The most frequently mentioned five dimensions by all participants were: mood (*N* = 52, 31.71%), physical activities (*N* = 48, 29.27%), work (*N* = 40, 24.39%), diet (*N* = 32, 19.51%), and vitality (*N* = 28, 17.07%). Significantly more healthy persons than patients reported mood (49.25 vs. 19.59%, *P* < 0.001), mindset (16.42 vs. 0.00%, *P* < 0.001), and self-care (11.94 vs. 2.06%, *P* = 0.016) characterizing good HRQoL, while more patients emphasized on work (4.48 vs. 38.14%, *P* < 0.001). When the five most important health dimensions were considered, there is generally no significant difference between subgroups defined by gender, age, or education in both healthy persons and patients (Table 3).

**Table 2:** Importance of each dimension by healthy persons and patients.

**Dimensions**	**Total (*****N*** **=** **164)**		**Healthy person (*****N*** **=** **67)**		**Patient (*****N*** **=** **97)**		***P*-value**
	**No**	**%**	**No**	**%**	**No**	**%**	
Mood	52	31.71	33	49.25	19	19.59	<0.001
Physical activities	48	29.27	20	29.85	28	28.87	0.892
Work	40	24.39	3	4.48	37	38.14	<0.001
Diet	32	19.51	14	20.90	18	18.56	0.710
Vitality	28	17.07	14	20.90	14	14.43	0.280
Pain	25	15.24	13	19.40	12	12.37	0.218
Sleep	17	10.37	5	7.46	12	12.37	0.311
Mindset	11	6.71	11	16.42	0	0.00	<0.001
Relationship	11	6.71	5	7.46	6	6.19	0.760
Self-care	10	6.10	8	11.94	2	2.06	0.016
Leisure activities	7	4.27	3	4.48	4	4.12	1.000
Cognition	4	2.44	3	4.48	1	1.03	0.306

**Table 3:** Difference in the importance of five most frequently mentioned dimensions among healthy persons and patients.

	**Mood (%)**	**Physical activities (%)**	**Diet (%)**	**Vitality (%)**	**Pain (%)**
**Healthy persons**					
***Gender***			^**^		
Male	53.13	21.88	31.25	21.88	21.88
Female	45.71	37.14	11.43	20.00	17.14
***Age***	^***^				
18–50	60.87	26.09	19.57	21.74	19.57
51+	23.81	38.10	23.81	19.05	19.05
***Education***					
High school or lower	48.78	34.15	19.51	24.39	21.95
College or higher	50.00	23.08	23.08	15.38	15.38
**Patients**					
***Gender***					
Male	39.66	27.59	20.69	22.41	15.52
Female	35.90	30.77	17.95	12.82	12.82
***Age***	^**^	*			
18–50	22.86	40.00	14.29	20.00	20.00
51+	46.77	22.58	22.58	17.74	11.29
***Education***					
High school or lower	28.57	28.57	21.43	28.57	14.29
College or higher	42.03	28.99	18.84	14.49	14.49

*** *Significant at 0.01 level;*

** Significant at 0.05 level; *Significant at 0.1 level.

## Discussion

Health dimensions identified in this study were similar to those found in previous studies of Chinese people through focus group discussion (6–12). However, some of those health dimensions identified from focus group discussion were not surfaced in the present study. For example, focus discussion revealed that freedom is a dimension of good HRQoL. Instead of reporting any mentioned dimensions of good HRQoL, this study focused on dimensions that were independently alluded to by at least two participants. The use of both qualitative and quantitative evidence minimized the risk of taking idiosyncratic views as commonality, making the findings more useful for informing culture-specific questionnaire adaptation and development.

Similar to the Western population, Chinese people considered mood, physical activities, work, and vitality as important dimensions of good HRQoL. However, vitality, or “jing shen” in Chinese, has different meanings between Chinese and Western cultures (13, 14). In Chinese culture, vitality mainly relates to mental health. On the contrary, in Western culture, vitality is related to both physical and mental health. Indeed, studies on the vitality scale of the SF-36 showed that the relationship between vitality and physical and mental health differed significantly between the Chinese and US populations (15–18). Since vitality is an important health dimension among Chinese but has different meanings from Western culture, attention should be paid to this dimension when adapting or developing HRQoL instruments for use among Chinese.

Different from Western people, Chinese people consider the ability to eat and drink as an important dimension of good HRQoL. Its importance in Chinese culture is evident by the customs that many Chinese acquaintances greet each other with “Have you had your meal?” when they bump into each other at noon or in the evening. In Chinese culture, eating and drinking ability is considered an indicator of good productivity and longevity. Therefore, when adapting HRQoL instruments developed in Western culture, the limitation of not having the diet dimension should be deliberated. When developing Chinese culture-specific HRQoL instruments, diet should be included as an important health dimension.

Significantly more patients emphasized work as a characteristic of good HRQoL. It is not surprising because the loss of working ability is an important concern to patients. The importance of mood and mindset was also different significantly between healthy and ill people. It is understandable that patients may have lowered their expectations and focus more on imminent concerns about their physical health and functions. In order to prevent ceiling or floor effect in measurement, health dimensions in HRQoL instruments for general and patient populations are better to be customized among Chinese. For the general population, dimensions representing mental health should be emphasized; for patient populations, dimensions concerning working ability are important.

The limitation of the study is that some potentially important health dimensions, such as sexuality, could not be identified from face to face interviews due to social desirability. Since talking about sex is taboo in Chinese culture, it is possible that respondents are unwilling to indicate it as a characteristic of good HRQoL in front of interviewers. In addition, the results should be interpreted with caution because study participants might not be representative of the general population.

## Conclusions

Through face to face interviews, this study not only identified 12 health dimensions representing good HRQoL by Chinese but measured their relative importance in Chinese culture. It appears that Chinese people have their culture-specific perception of dimensions characterizing good HRQoL. Diet and vitality are culture-specific dimensions related to health among Chinese. To better adapt or develop HRQoL instruments for Chinese, dimensions or items regarding diet are recommended to be included and disparities in the meaning of vitality between Chinese and Western cultures should be taken into account. In addition, mental health dimensions are important to Chinese generic HRQoL instruments and working ability dimensions are important to Chinese disease-specific HRQoL instruments. More research is needed to elucidate the health concepts held by the Chinese so that better HRQoL instruments can be developed for this population.

## Data Availability Statement

The raw data supporting the conclusions of this article will be made available by the authors, without undue reservation.

## Ethics Statement

The studies involving human participants were reviewed and approved by China Pharmaceutical University. The patients/participants provided their written informed consent to participate in this study.

## Author Contributions

ML, ZB, JZ, and NL contributed to conception and design of the study. ML and NL organized the database and wrote the first draft of the manuscript. ML, GL, PC, and NL performed the statistical analysis and wrote sections of the manuscript. All authors contributed to manuscript revision, read, and approved the submitted version.

## Conflict of Interest

The authors declare that the research was conducted in the absence of any commercial or financial relationships that could be construed as a potential conflict of interest.
